# Evaluating Nutrient-Based Indices against Food- and Diet-Based Indices to Assess the Health Potential of Foods: How Does the Australian Health Star Rating System Perform after Five Years?

**DOI:** 10.3390/nu12051463

**Published:** 2020-05-18

**Authors:** Sarah Dickie, Julie L. Woods, Phillip Baker, Leonie Elizabeth, Mark A. Lawrence

**Affiliations:** Institute for Physical Activity and Nutrition (IPAN), School of Exercise and Nutrition Sciences, Deakin University, Geelong 3220, Australia; j.woods@deakin.edu.au (J.L.W.); phil.baker@deakin.edu.au (P.B.); lelizabe@deakin.edu.au (L.E.); lawrence@deakin.edu.au (M.A.L.)

**Keywords:** nutrition policy, health star rating, nutrient profiling, NOVA, ultra-processed food, Australian Dietary Guidelines, front-of-pack label

## Abstract

Nutrient-based indices are commonly used to assess the health potential of individual foods for nutrition policy actions. This study aimed to evaluate the nutrient profile-informed Australian Health Star Rating (HSR), against NOVA and an index informed by the Australian Dietary Guidelines (ADGs), to determine the extent of alignment. All products displaying an HSR label in the Australian marketplace between June 2014 and June 2019 were extracted from the Mintel Global New Product Database, and classified into one of four NOVA categories, and either as an ADG five food group (FFG) food or discretionary food. Of 4451 products analysed, 76.5% were ultra-processed (UP) and 43% were discretionary. The median HSR of non-UP foods (4) was significantly higher than UP foods (3.5) (*p* < 0.01), and the median HSR of FFG foods (4) was significantly higher than discretionary foods (2.5) (*p* < 0.01). However, 73% of UP foods, and 52.8% of discretionary foods displayed an HSR ≥ 2.5. Results indicate the currently implemented HSR system is inadvertently providing a ‘health halo’ for almost ¾ of UP foods and ½ of discretionary foods displaying an HSR. Future research should investigate whether the HSR scheme can be reformed to avoid misalignment with food-and diet-based indices.

## 1. Introduction

Dietary risk factors are leading contributors to the global burden of disease [1]. Consequently, many policy actions aiming to promote healthy dietary behaviours and improve food environments have been recommended, including the restriction of marketing to children, food reformulation, economic incentives and disincentives, and interpretive front-of-pack nutrition labelling (FOPL) [2,3,4,5]. The implementation of nutrition policy requires an ability to assess the health potential of individual foods and beverages (herein referred to as food). The nutrition classification schemes (NCS) available for this assessment can be classified in accordance with the type of nutrition exposure on which their assessment indicators are based: nutrient-based indices; food-based indices; and diet-based indices [6,7,8].

Nutrient-based indices assess the health potential of foods according to the quantity of selected nutrients and/or other components. Nutrient-based indices are currently the most common form of NCS in policy action, and are generally operationalised through nutrient profiling (NP) [9,10,11,12]. Examples of policy actions applying a NP approach include the UK’s restrictions on marketing to children [13], the regulation of the eligibility of foods to display health claims in Australia [14], and 39 interpretive front-of-pack labels introduced worldwide [15]. The past decade has seen a proliferation of FOPLs implemented, including the Nutri-score label in France [16], the Multiple traffic lights (MTL) in the UK [17] and Ecuador [18], Warning Labels in Chile [19] and Israel [20], and the Health Star Rating (HSR) in Australia [21]. Evidence of associations between nutrient-based indices and non-communicable disease and obesity outcomes is currently lacking [15], although some predictive validation for the UK-created NP model exists [22,23,24,25].

Food-based indices expand the scope for nutrition classification, taking account of the structure (food matrix) and composition (ingredients) of foods. The NOVA classification scheme is a commonly used food-based index. Developed in Brazil in 2009, NOVA categorises food products into one of four categories (minimally processed (MP); processed culinary ingredients (PCI); processed (P); and ultra-processed (UP)) depending on the extent and purpose of industrial processing [26]. NOVA has been applied extensively in epidemiological research, with the reporting of significant associations between the proportion of UP foods consumed in the diet and adverse health outcomes, including cardiovascular disease (HR:1.12 for each 10% increment in UP foods in the diet) [27], cancer (HR_10%_:1.12) [28], depression (HR_10%_:1.21) [29], gastrointestinal disorders (OR_Q4 vs. Q1_:1.25) [30], and all-cause mortality (HR_Q4 vs. Q1_: 1.62) [31]. Furthermore, an experimental trial found diets consisting of UP foods increased ad libitum energy consumption, likely due to UP food’s low satiation and sensory properties [32]. In response, the Food and Agriculture Organization has published a report on using the NOVA classification system [26].

Diet-based indices assess the health potential of individual foods based on their inclusion in a healthy dietary pattern. Dietary patterns consider the amount, type, and diversity of foods consumed over the whole diet, and can be converted into indices for assessment purposes. For example, a binary system has been developed to interpret the Australian Dietary Guidelines (ADGs), whereby foods are either classified as recommended nutritious five food group foods or non-nutritious discretionary foods [33,34]. Evidence on healthy dietary patterns conclude associations between the DASH and Mediterranean diets and incidence of cardiovascular disease [35,36], adherence to the United States’ Healthy Eating Index and reduced risk of all-cause mortality [37], and plant-based diets and reduced risk of Type 2 Diabetes [38].

In June 2014, the Australian government endorsed the nutrient-based HSR, a voluntarily implemented FOPL, with a review planned after five years of implementation [39,40]. The HSR scheme uses a NP algorithm to assign eligible packaged foods a star rating in half-star increments between 0.5 and 5 based on the content of selected ‘beneficial’ and ‘risk’ nutrients and food components. Although the interpretive format of the HSR has been shown to be easily understood [41,42], the underlying algorithm has been subject to criticism. Concerns raised in stakeholder submissions on the performance of the system in mid-2017 included aspects such as the use of total instead of added sugars, the weighting of positive nutrients, whether the ratings should help differentiate discretionary foods from nutritious foods, and whether the system should reflect the level of food processing [43].

This study aims to evaluate the Australian HSR system, as implemented, against a food-based index, NOVA, and a diet-based index informed by the Australian Dietary Guidelines, to determine the extent of alignment. This research coincides with the completion of the five-year implementation period for the HSR system that was specified in 2015 [44]. The research extends two previous studies analysing food products displaying the HSR label released in Australia between 2014 and 2017 against the NOVA classification scheme [45] and the ADGs [46]. It is also particularly timely to evaluate the NP approach and evaluate its suitability for specific policy actions because the Codex Alimentarius Commission (Codex) is currently in the process of developing general guidelines for the use of NP in FOPLs [15].

## 2. Materials and Methods

The methods used in this research are based on those developed and implemented in earlier stages of this critical analysis investigation. Detailed description of these methods has been provided in previous papers reporting on this research [45,46].

### 2.1. Data Collection

Data were obtained from the Mintel Global New Product Database (GNPD), an extensive industry database collecting information on all new and updated packaged food and beverage products released on to the market worldwide. Systematic sampling was conducted of all new Australian food and beverage products displaying an HSR between the 27th of June 2014 and 30th of June 2019, spanning the first five years of the system’s implementation. The Mintel ‘baby foods’ and ‘alcoholic drinks’ categories were excluded from the sample as they are not eligible to display the HSR label. Detailed information on all products was extracted, including the number of health stars displayed, Mintel food category and sub-category, release date, product description, packaging images, nutrition composition, and ingredients list.

### 2.2. Nutrition Classification Schemes’ Categorisation of a Food’s Health Potential

Critical to the comparisons of the nutrient profile informed HSR system with the food-based index (NOVA) and the diet-based index (ADGs) was how individual foods were categorised in relation to their perceived health potential within each scheme. Here, the decision-making procedure for specifying categories and allocating individual foods to categories is described for the three chosen NCSs.

#### 2.2.1. HSR Classification Scheme

The design of the HSR classification scheme differs practically from the ADG and NOVA classification schemes in relation to how healthy and unhealthy foods are identified. The algorithm that underpins the HSR calculator produces a score that is converted to a star rating based on the type of product—general foods and beverages, dairy products and oils are scored on separate scales. Baseline points are calculated for risk nutrients: energy, saturated fat, sodium and total sugars, and modifying points are calculated for the proportion of fruit, nut, vegetable and legume (FVNL), fibre, and protein content. The HSR scheme uses a continuous scale and is intended to be applied for product comparisons within food categories. Therefore, unlike its predecessor the Nutrient Profiling Scoring Criterion (NPSC) [14], it does not specify a cut-off point to distinguish eligible (suitable to make a health claim) from ineligible foods. The design has led to difficulties in evaluating the scoring system, with no validated standard for determining what constitutes a rating cut-off point for distinguishing between a healthy and unhealthy product. Yet, as an interpretive FOPL, the effectiveness of the HSR scheme is dependent on consumers’ interpretation of its visual component, and its ability to modify the health perception of a food product [47]. However, there is currently no evidence on the rating cut-off point at which this perception is modified on the HSR’s 10-point scale.

In the absence of any evidence-informed justification to the choice of HSR cut-off value, this study selected a ‘logic-informed’ HSR cut-off of 2.5 to distinguish healthy categories from unhealthy categories, because 2.5 is a ‘healthy pass’ on a 5-star scale. Consequently, the ADGs recommended five food group foods would be expected to display an HSR at or above 2.5 stars. Conversely, consumption of discretionary foods is not recommended as part of a healthy diet, and therefore they would be expected to display a star rating of 2 stars or below, or a ‘fail’. Similarly, NOVA’s least processed category, unprocessed and minimally foods, would be expected to display an HSR at or above 2.5 stars and ultra-processed foods would be expected to display 2 stars or below. The proportion of products falling above and below a 3.5 cut off was also calculated in this study to enable comparison of results with the findings from other studies that had adopted this cut-off level to demarcate nutritious foods from discretionary foods [48,49,50,51,52].

#### 2.2.2. NOVA Classification Scheme

The NOVA classification scheme was selected to represent food-based classification schemes. NOVA food categories were based on the description outlined by Monteiro et al. [53], which specifies foods into four groups: unprocessed or minimally processed (MP) foods; processed culinary ingredients (PCI); processed (P) foods; and ultra-processed (UP) foods.

Epidemiological evidence from NOVA studies [26] highlights that it is primarily the ability to distinguish UP foods from foods within the other three categories, i.e., a binary approach, that is the most relevant point of differentiation between ‘healthy’ and ‘unhealthy’ foods. UP foods were identified based on the presence of a processed food substance or cosmetic additive in the ingredients list. Research by Machado et al. [54] was referred to for the identification of UP-characterising ingredients, and the processed status of novel ingredients was discussed amongst all authors until a consensus was reached.

#### 2.2.3. ADG Classification Scheme

The ADGs were selected to represent a diet-based classification scheme. The classification of ADG food categories was based on those depicted in the 2013 Australian Guide to Healthy Eating (AGTHE) [55] and the ADGs Educator Guide [56]. Three ADG coding categories were specified: (i) FFG foods (fruit; vegetables; grain foods; meat, eggs, tofu, nuts, seeds, and legumes; milk, yoghurt, cheese, and alternatives; and mixed meals consisting mostly of FFG foods); (ii) Discretionary foods; and (iii) A small number of ‘other’ foods (culinary ingredients; formulated supplementary foods; and water).

Discretionary foods were identified using a procedure developed by the Australian Bureau of Statistics (ABS) for the analysis of the 2011–2012 Australian Health Survey (AHS) [33]. The ABS procedure involved two steps: First, the ABS developed ‘Principles for Identifying Discretionary Foods’; and second, a detailed Discretionary Food List, wherein discretionary items were flagged in the AUSNUT 2011–2013 food composition database. A transparent and documented procedure was created for products difficult to classify. Where there were difficulties in classification, all authors reached a consensus decision based on the ingredients list and food purpose.

When product categorisation for both the ADG and NOVA schemes was uncertain, a conservative approach to decision making was adopted in which the decision defaulted to categorising the food product into the FFG category, or the NOVA category with the lower level of processing. Two researchers classified all products independently, and if disagreement occurred, consensus was reached by discussion among all researchers. Two co-researchers, who were not involved in initial classification, undertook validation of the ADGs and NOVA categorisation decision by cross-checking a 5% random selection of the total sample.

### 2.3. Statistical Analysis

All statistical analyses were conducted in STATA version 16 [57]. Descriptive statistics including HSR frequency, median, range, and interquartile range were produced for the overall sample, the four NOVA categories and ADG categories collapsed into FFG and discretionary foods. The number of products in each NOVA and ADG category falling above and below both the 2.5 and 3.5 HSR cut-offs was calculated. The HSR frequencies and medians were also calculated by implementation year for the total sample and each category. As the HSR system was endorsed on the 27th of June 2014, each implementation year was defined as a calendar year starting on the 27th of June and ending on the 26th of June. Descriptive analysis by the food or beverage Mintel category level and sub-category level was performed for the overall sample and each NOVA and ADG category. Mintel groups foods into 16 food categories (excluding baby food), and seven drinks categories (excluding alcoholic beverages), with each category further divided into granular sub-categories. Mintel defines these categories based on product similarities, and these do not correspond with the NOVA and the ADG criteria (for example products in the Mintel category “water” may be classified as UP or discretionary, as they may be sweetened or highly processed types of water).

Mann-Whitney U tests were performed to determine differences between the FFG and discretionary HSR medians, and between the HSR medians of UP foods and the three non-UP food categories combined.

## 3. Results

**Figure 1:**
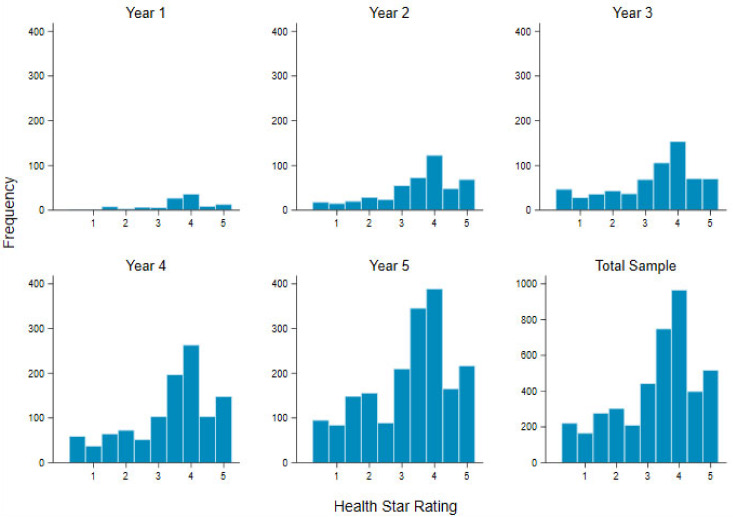
Health Star Rating frequency by implementation year and total sample.

A total of 4251 new food and beverage products were released displaying an HSR in the first five years of the HSR systems’ implementation, representing 17.6% of all new products released into the Australian marketplace during the same time period (6 June 2014–30 June 2019, inclusive). A progressively larger number of products displaying the HSR were released each year across the five years, but the distribution of HSR rating frequencies did not vary considerably by implementation year (Figure 1). The most frequently displayed rating across the five years was 4 stars, representing 22.7% (n = 965) of all products, followed by 3.5 stars, representing 17.6% (n = 749) (Figure 1). The rating of 1 star was displayed least frequently, on 3.9% (n = 166) of products. The median HSR for the total sample was 3.5 stars, although was higher at 4 stars in the first two years of implementation (Table 1). A total of 22.8% of products scored <2.5, with the percentage rising slowly each year, 77.6% scored ≥2.5, and 61.8% scored ≥3.5, with the percentages decreasing each year.

The Mintel category most frequently displaying the HSR label was snacks at 15.6% (n = 657), followed by bakery products at 13.5% (n = 574), and processed fish, meat and egg products at 12.1% (n = 516). The proportion of products displaying HSRs for snacks, bakery products, and processed fish and egg products is slightly higher than the proportion of these products released during the same time period, at 12.6%, 12.7%, and 8.6%, respectively. The sub-category of snack/cereal/energy bars had the highest proportion of snack products displaying an HSR (n = 224), and the median HSR for these products was 4 stars (Table S1).

### 3.1. Comparison of the HSR System with NOVA

Most products in the total five-year sample (76.5%) were classified as UP (n = 3253), with 12.7% of products as MP (n = 538), 9.6% as P (n = 407), and only 1.2% as PCI (52) (Table 2). The median star rating for MP, P, non-UP combined, and UP food products was 4.5, 4, 4, and 3.5, respectively. PCIs had a relatively lower median of 2 stars, although this varied between implementation years, with medians of 3, 3.5 and 4.5 observed in later years. The median star ratings for each NOVA category increased over time for MP foods and PCIs and decreased over time for UP foods and non-UP foods combined, but remained stable for P foods. Although the median HSR for each category of NOVA was ≥2.5 (healthy pass), there were significant differences between the median HSR of the UP category (median 3.5) and that of MP (median 4.5), P (median 4) and non-UP groups combined (median 4) (all *p* < 0.01). For UP foods, 73.0% scored an HSR ≥ 2.5, and 55.3% scored an HSR ≥ 3.5, compared to 91.0% and 83.1% for non-UP foods combined.

**Figure 2:**
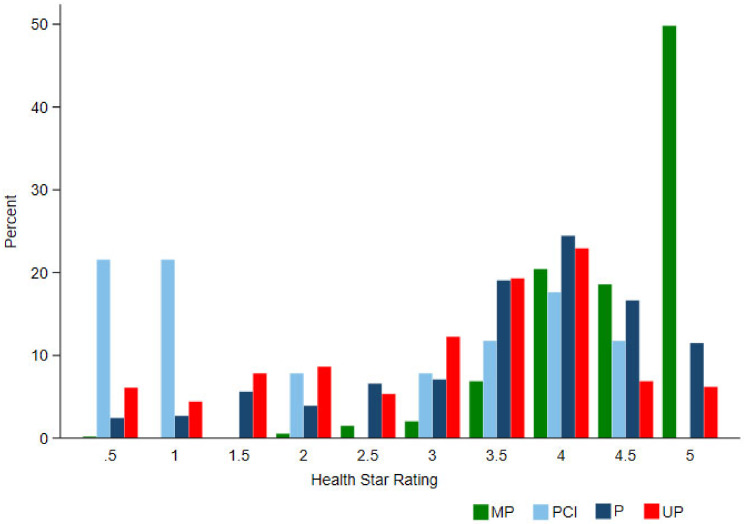
Percent HSR frequency within NOVA categories compared for the total sample.

**Figure 3:**
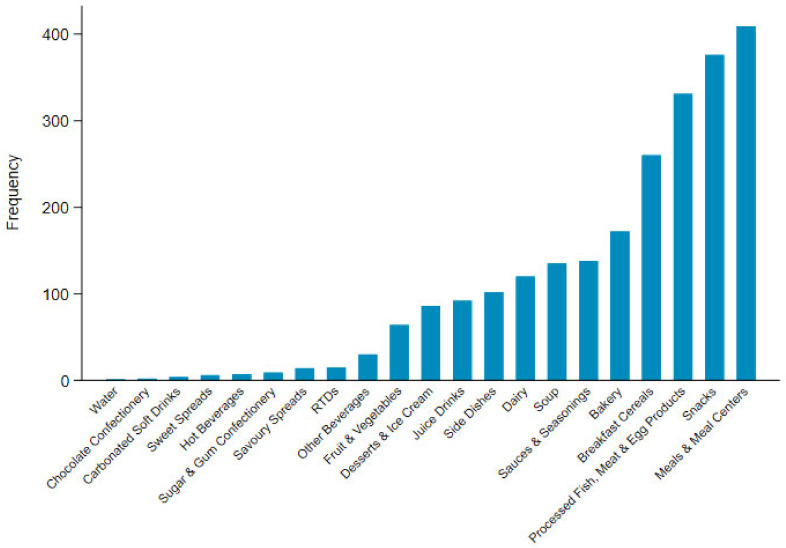
Frequency of UP products scoring a Health Star Rating ≥2.5 by Mintel category.

The distribution of star ratings for MP food products was strongly skewed towards higher HSRs (Figure 2), with only four products receiving a rating under 2.5 stars (Table 2). These products were sour cream at 0.5 stars, and three snack mixes consisting of seeds and nuts, all of which received two stars. For UP foods, meals and meal centres (e.g., instant noodles and prepared meals) (n = 409), snacks (e.g., energy bars and potato chips) (n = 376), and processed fish, meat and egg products (n = 331) had the highest frequencies of foods scoring ≥2.5 and represented 47% of all of the UP foods with these scores (Figure 3).

### 3.2. Comparison of the HSR System with the Australian Dietary Guidelines

The majority of products in the sample (55.5%) were classified as FFG foods (n = 2360), and of these, the highest frequencies were observed for grains (n = 578) and meat/legumes/nuts/seeds/eggs (n = 648) (Table 3). The median HSR rating for FFG foods was 4 stars, significantly higher than the median for discretionary foods at 2.5 stars (*p* < 0.01). Fruit and vegetables had the highest median HSR of all food groups, both at 4.5 stars. The median HSR for FFG foods remained at 4 stars for each implementation year; however, the median HSR for discretionary foods decreased over time, from 4 stars in the first year, to 2 stars in the fifth year (Table 3).

**Figure 4:**
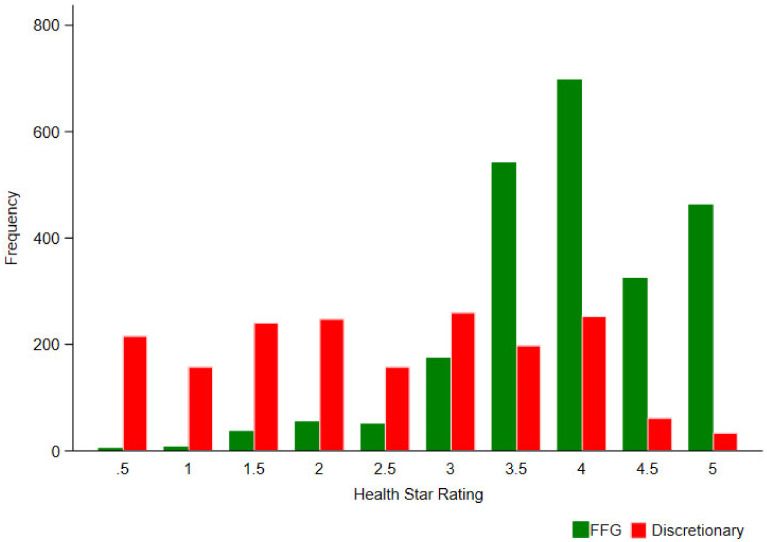
Health Star Rating frequency for the total sample: Five food group (FFG) foods compared to discretionary foods.

**Figure 5:**
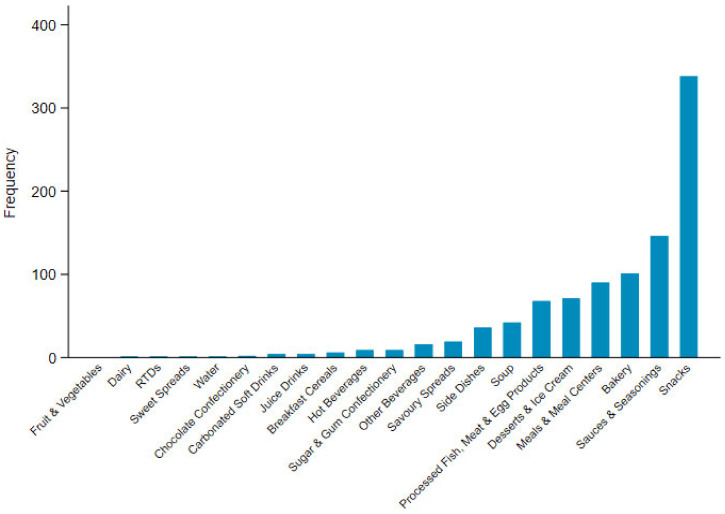
Frequency of discretionary products scoring a Health Star Rating ≥2.5 by Mintel category.

The distribution of star ratings for FFG foods was strongly skewed towards higher star ratings (Figure 4), and the majority scored ≥2.5 stars (95.5%). The star ratings for discretionary foods were evenly distributed along the 10-point scale (Figure 4), and 52.8% scored ≥2.5 stars. Of the Mintel categories, Snacks had the largest proportion of discretionary products scoring ≥2.5 stars, at 35% (Figure 5).

## 4. Discussion

This research compared the HSR system, a FOPL informed by a nutrient-based index, with a food-based index, NOVA, and a diet-based index informed by the ADGs. Uptake of the HSR in new products increased by 235% in the last two years of implementation, although the distribution and frequency of star ratings did not change from the previous three years of findings, with a HSR median of 3.5 and a clustered frequency at 3.5 and 4 stars [46]. The HSR’s alignment with NOVA and the ADGs, particularly the observed misalignment with UP and discretionary categories, also did not differ from earlier findings [45,46]. These results are not unexpected considering no changes have been made to the underlying algorithm during these time periods, despite review of the system after two and five years, and concerns raised by stakeholders [43].

The median HSRs for MP, P and UP foods (4.5, 4, 3.5) did not change from those reported for the three-year analysis [45], with a clear gradient in HSRs observed alongside increasing level of processing. Few studies have analysed the HSR system against level of processing, and only the authors’ previous study calculated median star ratings of NOVA categories for comparison [45]. The difference in HSR medians between the ‘healthy’ (non-UP foods) and ‘less healthy’ (UP foods) categories of NOVA was significant (*p* < 0.01). This difference can indicate a certain level of discrimination between ‘healthy’ and ‘less healthy’ foods, although for NOVA this discrimination is negligible with only a 0.5-star difference, and both medians in the ‘healthy pass’ range. With such a high proportion of UP foods scoring at or above 2.5, it is clear that a comparison of medians alone is not sufficient to provide an assessment of delineation between categories. Thus, significant differences in medians should not be solely relied on as an indication of alignment with a food-based index.

The median star ratings observed for both FFG foods (4 stars) and discretionary foods (2.5 stars), and the significant difference between medians (*p* < 0.01), also did not differ from our previous three-year analysis [46]. The median for FFGs aligns with four previous studies analysing the HSR against ADGs, which all observed a median of 4 stars [48,50,58], and is slightly above one study observing a median of 3.5 stars [49]. However, the median for discretionary foods was higher than those reported in all previous studies (excluding the authors’), which observed medians of 2 stars [48,49,50,58]. Although, differences in medians should not be the sole indicator of alignment between indices. For example, HSR frequencies for discretionary foods were highly dispersed over the 10-point scale in the current research, with the frequency of products scoring 3 and 4 stars being similar to the frequency of products scoring 1.5 and 2 stars (Figure 4).

A high degree of alignment was observed between the HSR and the ‘healthy’ categories based on the predetermined cut off (HSR ≥ 2.5). In contrast, a high degree of misalignment was observed for the ‘less healthy’ categories, UP and discretionary foods, at 73% and 53%, respectively. The appropriate cut-off point at which a food is determined ‘healthy’ on the HSR scale is unresolved. One of the earliest analyses of the HSR system reported that the majority (79%) of recommended ADG five food group foods in New South Wales public settings scored an HSR ≥ 3.5, and 86% of discretionary foods scored an HSR < 3.5 [49]. Subsequently, an HSR cut off value of 3.5 has been assumed to be a valid standard and adopted in many research activities [48,50,51,52], and dubiously argued to provide a reference standard for annual surveys comparing countries’ food supplies [59]. Menday et al. reported 16.3% disagreement with FFG foods and 27.5% disagreement with discretionary foods [50], and Jones et al. reported 9.5% disagreement with FFG foods, and 17.4% disagreement for discretionary foods, both studies concluding broad alignment with the ADGs. When applying the 3.5 cut off in this research, the misalignment between the HSR and ‘less healthy’ categories was still high, 55.3% for UP, and 29.9% for discretionary, significantly higher than the results reported for discretionary foods by Jones et al. The higher cut off also resulted in decreased alignment with ‘healthy’ categories, up to 10% less FFG foods qualified, thus the 2.5 cut-off appears to have greater alignment for ‘healthy’ foods.

There is no scientific basis to selecting a cut off of 3.5 stars as an appropriate value at which to distinguish healthy foods. The adopted cut off level’s relatively high calibration may serve to capture the majority of five food group foods; however, it also has the effect of masking the extent of misalignment of the star rating on UP and discretionary foods. A qualitative study found participants generally considered a product healthy if it displayed 3 or more stars, and unhealthy if displaying 2 or less [60]. This highlights the ambiguity present in the mid-range of the scale, as consumers convert scales to binary cues (healthy/unhealthy), and the HSR does not provide a clear point at which to make this conversion [61]. Curiously, the HSR formal five-year review concluded that the system aligned with the ADGs, but was based on research reporting on a 3.5 HSR cut off [40]. More research is needed into consumers’ perceptions on the acceptable cut off to distinguish ‘healthy’ foods from ‘less healthy’ foods.

The lack of alignment between the three indices can be partly explained by technical aspects associated with their design, e.g., the HSR scheme design defines total sugar and not added sugar as a risk ingredient, whereas the ADGs refer explicitly to foods with added sugars as discretionary foods. However, design characteristics alone are unlikely to account for the extent of misalignment. The science informing each index represents different understandings of the associations between nutrition exposures and health outcomes. The nutrition science informing the HSR is based on an understanding that a food’s health potential can be predicted by simply summing the amounts of its individual parts (nutrients). However, there are over 26,000 distinct bio-active components present in food [62] and yet most nutrient profiling approaches are based on calculations involving just a handful of these components. Conversely, the nutrition science informing NOVA and the ADGs is based on the principle that a food’s health potential is more than the sum of the nutrients it contains because a nutrient’s absorption can be affected by the presence of other nutrients/components in the food as well as the physical structure of the food matrix [63,64].

A further limitation with the HSR system is the positive orientation of its ‘health’ star message. The ADGs and the FAO state that discretionary foods and UP foods, respectively, are not essential components of the diet [26,34]. Yet, all HSRs provide a positive score, i.e., the minimum is 0.5 health stars, regardless of whether a food is discretionary or a UP food. There is concern that this can provide a ‘health halo’ to foods that the population should be consuming less frequently [65]. In Australia, over half of the packaged food supply is composed of discretionary and UP foods [51]; discretionary foods are price promoted twice as often [66] and advertised more frequently than FFG foods [67,68], and most high-market-share UP foods display some form of marketing on the packaging [69]. This marketing environment undoubtedly influences Australia’s dietary intake, where 35% of the average daily energy intake is consumed from discretionary foods (ABS), and 42% from UP foods [54]. An effective FOPL should discourage UP and discretionary foods’ selection and consumption [47,70]. Yet, our results highlight that too many are displaying relatively high HSRs, and thus the system, in its current implementation, may not be effectively countering the marketing or decreasing the consumption of these products. Furthermore, it reduces trust in the FOPL system; for example, Pelly et al. reported that consumers were sceptical about the HSR as it often resulted in too high star ratings being displayed on ‘junk foods’ [65].

Only two other studies have previously analysed the same three nutrient-based, food-based and dietary-based indices [51,71]. Results obtained by Pulker et al. were moderately consistent with our observations, finding that 55% of UP foods and 33% of discretionary foods scored an HSR ≥ 2.5, likely due to comparable methodology, although only supermarket own brands were analysed [71]. Conversely, Crino et al. found 19% of ‘highly processed foods’, and 10% of discretionary foods received an HSR ≥ 3.5 [51]. These results are comparatively lower than our results of 55% for UP foods and 30% for discretionary foods when assessed at the 3.5 cut off. The differences observed could be due to a number of other factors; Crino et al. analysed all packaged products available in the Australian marketplace (using the Foodswitch database), FVNL (fruit, vegetable, nut and legume content) information had to be estimated for the HSR calculation for an unknown number products, and possible differences in classification methodology. For example, an adapted version of NOVA was utilised and details on how discretionary foods were identified was not presented. Thus, research on nutritional classification indices is difficult to compare without the use of rigorous and consistent methodologies between studies.

As the NOVA classification scheme identifies UP foods based on the presence of one or more additives, a heterogeneous mix of food types were observed receiving high star ratings. For example, in the bakery category, products ranged from pancake mixes and sweet breakfast biscuits, to foods that appear ‘healthy’, such as whole grain barley wraps, a product that contains extracted wheat fibre and wheat gluten, thickeners, glycerol, emulsifiers and dextrose. The interaction of these substances in a food, and how they are metabolised has unknown consequences on long-term health, and the simplicity inherent in NP is unable to identify these ‘food-like’ products. For discretionary foods, most misalignment was seen in the snacks category, and within this snack/cereal/energy bars featured prominently. Most of these products contain added sugar, usually in the form of glucose syrup, although their make-up allows the addition of nuts, seeds, protein powders and inulin, all of which help manufacturers to garner a higher HSR and market their products as ‘healthy’.

In enabling the promotion of UP and discretionary foods, the HSR system is failing a key requirement of an effective food labelling policy—to not be misleading [72]. Our results indicate manufacturers can manipulate the composition of their food products to meet the ‘healthy’ requirements of the nutrient-based algorithm and use the HSR label as another marketing tool for highly profitable ‘junk foods’. For example, ‘Nutri-Grain To Go Banana & Honey Smash Protein Squeezer’, a product classified as both discretionary and UP, receives 3.5 health stars, and contains banana puree, protein isolates, and vegetable fibres, all of which may contribute to a more favourable rating. It is thereby unlikely to be a coincidence that food manufacturers generally support ‘softer’ regulatory policies informed by NP, such as positively orientated FOPLs and reformulation strategies [73]. Food companies may not consider these actions as a significant threat to profits as they create opportunities for marketing differentiation [73].

Although the aim of this study was to evaluate a nutrient-based index, the results also indicate a degree of misalignment between the food- and dietary-based indices. NOVA classified substantially more of the sample into the ‘less healthy’ category compared to the ADGs, and the UP median at 3.5 stars was well above the discretionary median at 2 stars. This misalignment is not surprising considering the ADGs do not incorporate the concept of processing. Whether or not the ADGs would benefit from the incorporation of NOVA principles, following the lead of South American nations [74,75], is outside the scope of this paper, and requires further research.

There is limited research comparing other nutrient-based indices with food-or dietary-based indices. The NP model developed by the Food Standards Agency in the UK, ‘Ofcom’, has been compared to NOVA and reported a 79.4% agreement [76] and a study testing convergent validity against the UK’s dietary guidelines found good agreement (k = 0.69) [77]. Both of these studies concluded consistency between indices, although there is no standard for how results should be interpreted to make these conclusions. The current study found the implemented Health Star Rating system is misaligned with NOVA and the ADGs, logically based on the majority of ‘less healthy’ foods failing on the 10-point scale. The limited predictive validation, and findings from this research, indicate the need for caution not only on the continued use of NP models for nutrition policy actions, but also for the evaluation of national food supplies [59].

This is the first study to conduct an independent analysis of the HSR implementation to coincide with the completion of its five-year implementation period. The research is novel as it is one of only three studies comparing a nutrient-based index with food- and dietary-based indices. This was a notable achievement because the novel nature of the rapidly evolving packaged food supply, made classification using NOVA and the ADGs challenging in some circumstances. Ingredients could not always be easily identified as UP-characterising, nor could foods be easily identified as discretionary, mainly due to the limitations in the ABS method described previously [46]. However, these challenges were addressed by adhering to strict procedures for categorisation decisions. For instance, a rigorous process was used for the classification of NOVA and the ADGs, with stepwise rules developed (Scheme S1) and strictly adhered to, and discussion and consensus required amongst three researchers for difficult to classify products. Furthermore, this is the only study to sample observations in the marketplace to evaluate how the HSR is performing in practice as distinct from the reliance on modelling assumptions to estimate the potential market uptake and scoring profiles.

Nevertheless, there are several limitations with the analysis. The Mintel GNPD collects data on newly released products; therefore, our sample may not necessarily capture all food and beverage products displaying the HSR label in the marketplace if the label was added to a pre-existing product. However, the sample at 4251 is likely to represent nearly half of those products currently in the Australian marketplace displaying any form of the HSR label. By comparison, in mid-2018, 10,300 products were thought to display the HSR label, although this figure represents products displaying both the star rating graphic, and those displaying only the energy icon [78].

The current study reports on the HSR implementation in practice, as a voluntary system, not a modelled mandatory system. The HSR’s voluntary implementation potentially allows manufacturers to selectively display the label on products scoring favourable star ratings. The higher median observed for discretionary foods in this study compared to studies analysing a mandatory system could be an indication of selective uptake by manufacturers. Jones et al. calculated HSR’s for a large database of Australian food products whether the symbol was displayed or not, observing a median of 2 stars for discretionary foods [48]. Furthermore, the two leading supermarkets in Australia have made commitments on HSR uptake for all own brand products [71], and therefore have less incentive to selectively display the HSR label. This is the conclusion of a recent study, with discretionary branded foods displaying a higher median HSR (4 stars), than discretionary supermarket own brands (3.5 stars) [79].

The NOVA scheme has attracted some criticism, which has focused mainly on the limitations of the broad and simple definition of ultra-processed foods [80]; however, criticisms were found to be mainly from authors with links to the ultra-processed industry [81]. Although an accurate example of a dietary-based index for the aims of this research, the ADGs were not designed to be used as a binary classification system at the food-based level. The ADG’s purpose and level of complexity make it inherently difficult to systematically classify individual foods in a binary fashion. The ABS has devised a method to identify discretionary foods [33], as used in this research, but it lacks the detail necessary to classify the array of novel packaged products currently available in the Australian marketplace with certainty, and further procedures had to be applied for classification. Thus, it is likely the classification of discretionary foods has differed between studies. Australia’s National Health and Medical Research Council are currently revising the methodology for identifying discretionary foods, and thus, classification difficulties may be reduced in any future analyses [82].

This research did not assess the market share of HSR-labelled products or the population dietary intake that may be influenced by the label; therefore, further research extending this work would be worthwhile.

## 5. Conclusions

The design and implementation of the current Health Star Rating system is misleading consumers about the healthiness of many foods by inadvertently providing a ‘health halo’ for almost three quarters of ultra-processed foods and half of discretionary foods displaying the symbol. Generally, the nutrient profiling approaches used to inform nutrition policy actions internationally are conceptually and technically similar to that which underpins the Health Star Rating system, and therefore this research has implications beyond Australia’s Health Star Rating system. Future research should investigate how nutrition science principles, including that a food’s health potential, is determined by more than sum of the nutrients it contains, can feature in the design of the Health Star Rating system, and other nutrient profiling systems, to avoid their misalignment with food- and diet-based indices.

## Figures and Tables

**Table 1:** Descriptive statistics for total sample by implementation year.

	n (%)	Median HSR	Range	IQR	n HSR ≤ 2 (%) *	n HSR ≥ 2.5 (%) *	n HSR ≥ 3.5 (%) *
**Year 1**	113 (2.7)	4	0.5–5	0.5	15 (13.3)	98 (86.7)	85 (75.2)
**Year 2**	475 (11.2)	4	0.5–5	1	82 (17.3)	393 (82.7)	314 (66.1)
**Year 3**	662 (15.6)	3.5	0.5–5	1.5	155 (23.4)	507 (76.6)	401 (60.6)
**Year 4**	1100 (25.9)	3.5	0.5–5	1.5	234 (21.3)	866 (78.7)	711 (64.6)
**Year 5**	1901 (44.7)	3.5	0.5–5	2	484 (25.5)	1417 (74.5)	1118 (58.8)
**Total**	4251	3.5	0.5–5	1.5	970(22.8)	3281(77.2)	2629 (61.8)

*n* number of products, HSR Health Star Rating, IQR interquartile range, * percentage within implementation year.

**Table 2:** Descriptive statistics of HSR by NOVA category and implementation year.

NOVA	*Year*	*n* (%)	HSR Median	HSR Range	IQR	*n* HSR ≤ 2 (%) *	*n* HSR ≥ 2.5 (%) *	*n* HSR ≥ 3.5 (%) *
**MP**	Year 1	10	4	3–5	1.5	0 (0)	10 (100.0)	9 (90.0)
	Year 2	44	5	2–5	0.5	1 (2.3)	43 (97.7)	42 (95.5)
	Year 3	80	4.5	0.5–5	1	1 (1.2)	79 (98.8)	76 (95.0)
	Year 4	151	5	2–5	1	1 (0.7)	150 (99.3)	146 (96.7)
	Year 5	253	4.5	2–5	1	1 (0.4)	252 (99.6)	242 (95.7)
	**Total**	**538 (12.7)**	**4.5**	**0.5–5**	**1**	**4 (0.75)**	**534 (99.25)**	**515 (95.72)**
**PCI**	Year 1	1	0.5	0.5–0.5	0	1 (100.0)	0	0
	Year 2	0	-	-	-	-	-	-
	Year 3	10	**	0.5–4	0.5	8 (80.0)	2 (20.0)	1 (10.0)
	Year 4	14	3.5	1–4.5	2	5 (35.7)	9 (64.3)	8 (57.1)
	Year 5	182	3	0.5–4.5	3	12 (6.6)	170 (93.4)	168 (92.3)
	**Total**	**51 (1.2)**	**2**	**0.5–4.5**	**3**	**26 (51.00)**	**25 (49.0)**	**21 (41.2)**
**P**	Year 1	9	3.5	2.5–4.5	1.5	0 (0)	9 (100.0)	6 (6.7)
	Year 2	53	4	0.5–5	1	8 (15.1)	45 (84.9)	44 (83.0)
	Year 3	66	4	0.5–5	1	8 (12.1)	58 (87.9)	55 (83.3)
	Year 4	97	4	0.5–5	0.5	9 (9.3)	88 (90.7)	75 (77.3)
	Year 5	182	3.5	0.5–5	1.5	35 (19.2)	147 (80.8)	111 (61.0)
	**Total**	**409 (9.6)**	**4**	**0.5–5**	**1.5**	**60 (14.7)**	**347 (85.3)**	**293 (71.6)**
**Non-UP combined**	Year 1	20	**	0.5–5	1	1 (5.0)	19 (95.0)	15 (75.0)
	Year 2	97	4.5	0.5–5	1	9 (9.3)	88 (90.7)	86 (88.7)
	Year 3	157	4.5	0.5–5	1.5	17 (10.8)	140 (89.2)	133 (84.7)
	Year 4	262	4.5	0.5–5	1.5	15 (5.7)	247 (94.3)	229 (87.4)
	Year 5	462	4	0.5–5	1.5	48 (10.4)	414 (89.6)	366 (79.2)
	**Total**	**998**	**4**	**0.5–5**	**1.5**	**90 (9.0)**	**908 (91.0)**	**829 (83.1)**
**UP**	Year 1	93	4	0.5–5	0.5	14 (15.1)	79 (84.9)	70 (75.3)
	Year 2	378	3.5	0.5–5	1.5	73 (19.3)	305 (80.7)	228 (60.3)
	Year 3	506	3.5	0.5–5	2	138 (27.3)	368 (72.7)	269 (53.1)
	Year 4	838	3.5	0.5–5	2	219 (26.1)	619 (73.9)	482 (57.5)
	Year 5	1440	3.5	0.5–5	2	436 (30.3)	1004 (69.7)	753 (52.3)
	**Total**	**3253 (76.5)**	**3.5 ^δ^**	**0.5–5**	**2**	**880 (27.00)**	**2375 (73.0)**	**1800 (55.3)**
**Total**		**4251**	**3.5**	**0.5–5**	**1.5**	**970 (22.8)**	**3281 (77.2)**	**2629 (61.8)**

*n* number of products, MP unprocessed and minimally processed, PCI processed culinary ingredients, P processed, UP ultra-processed, (%) * percentage of products within each NOVA category, ** median in a 0.5 increment could not be calculated, ^δ^ median significantly different to median of MP and P groups, and median of non-UP groups combined (Mann-Whitney U test, *p* < 0.01).

**Table 3:** Descriptive statistics of HSR by Australian Dietary Guidelines food category and implementation year.

ADG		n (%)	HSR Median	HSR Range	IQR	n HSR ≤ 2 (%) *	*n* HSR ≥ 2.5 (%) *	*n* HSR ≥ 3.5 (%) *
**FFG Foods**	Year 1	77	4	2.5–5	0.5	0	77	61
	Year 2	267	4	0.5–5	1	11	256	226
	Year 3	368	4	0.5–5	1	15	353	316
	Year 4	608	4	0.5–5	1	28	580	535
	Year 5	1039	4	1–5	1	51	988	880
	**Total**	**2359 (55.5)**	**4**	**0.5–5**	**1**	**105 (4.5)**	**2254 (95.5)**	**2028 (86.0)**
**Disc Foods**	Year 1	35	4	0.5–5	2.5	15	20	13
	Year 2	204	3	0.5–5	2	71	133	84
	Year 3	286	2.5	0.5–5	2.5	140	146	78
	Year 4	471	2.5	0.5–5	2	204	267	160
	Year 5	832	2	0.5–5	2	433	399	212
	**Total**	**1828 (43.0)**	**2.5 ^δ^**	**0.5–5**	**2**	**863 (47.2)**	**965 (52.8)**	**547 (29.9)**
**Grains**		578 (13.6)	4	1.5–5	3	16 (2.8)	562 (97.2)	502 (86.9)
**Fruit**		272 (6.4)	4.5	1.5–5	1	3 (1.1)	269 (98.9)	250 (91.9)
**Vegetables**		295 (6.9)	4.5	3–5	1	0	295 (100.0)	285 (96.6)
**MLNSE**		647 (15.2)	4	1–5	1	53 (8.2)	594 (91.8)	554 (85.6)
**Dairy/alternatives**		225 (5.3)	4	0.5–5	1.5	29 (12.9)	196 (87.1)	141 (62.7)
**Mixed foods**		342 (8.1)	3.5	0.5–5	0.5	4 (1.2)	338 (98.8)	296 (86.6)
**Culinary**		37 (0.9)	3.5	2–5	1	2 (5.4)	35 (94.6)	27 (73.0)
**FSF**		18 (0.4)	5	4–5	0.5	0	18 (100.0)	18 (100.0)
**Water**		9 (0.2)	5	5–5	0	0	9 (100.0)	9 (100.0)
**Total Sample**		**4251**	**3.5**	**0.5–5**	**1.5**	**970 (22.8)**	**3281 (77.2)**	**2629 (61.8)**

ADG Australian Dietary Guidelines, *n* number of food products, HSR Health Star Rating, IQR interquartile range, FFG five food group, * (%) of products within food group, ^δ^ HSR median significantly different to FFG median (Mann-Whitney ***U*** test, *p* < 0.01).

## Supplementary Materials

The following are available online at https://www.mdpi.com/2072-6643/12/5/1463/s1, Table S1: Descriptive statistics by Mintel categories, Scheme S1: Australian Dietary Guidelines classification rules.
