# Supplementary material for: Evaluating Nutrient-Based Indices against Food- and Diet-Based Indices to Assess the Health Potential of Foods: How Does the Australian Health Star Rating System Perform after Five Years?
Source: Nutrients. 2020 May 18;12(5):1463. doi: 10.3390/nu12051463 (PMC7284529; doi:10.3390/nu12051463)
Supplement: Supplementary file 1 [file nutrients-12-01463-s001.pdf]

Table S1. Descriptive statistics for the Health Star Rating by Mintel Category and Sub-category.

Category	Sub-category	*n*	HSR Median	Range	IQR
**Bakery**		**574**	**2**	**0.5-5**	**2**
	Sweet Biscuits	185	1	0.5-4.5	1
	Baking Ingredients & Mixes	88	2.5	0.5-5	2.5
	Bread & Bread Products	97	3.5	0.5-5	1
	Savoury Biscuits/Crackers	74	2.75	0.5-5	1.5
	Cakes, Pastries & Sweet Goods	130	1.5	0.5-3.5	0.5
**Breakfast Cereal**		**324**	**4**	**1.5-5**	**0.5**
	Cold Cereals	243	4	1.5-5	0.5
	Hot Cereals	81	4.5	3-5	1
**Carbonated Soft Drinks**		**25**	**1**	**1-5**	**1**
**Chocolate Confectionary***		**46**	**0.5**	**0.5-3**	**0**
**Dairy**		**240**	**3.5**	**0.5-5**	**2**
	Spoonable Yoghurt	33	4	1.5-5	2
	White Milk	44	4	3.5-5	1
	Hard Cheese & Semi-Hard Cheese	25	2.5	0.5-5	1.5
	Soft Cheese & Semi-soft Cheese	17	2.5	0.5-5	2.5
	Processed Cheese	8	2	0.5-3.5	1
	Flavoured Milk	36	4	2-5	1.5
	Drinking Yoghurt & Liquid Cultured Milk	10	3.5	0.5-4.5	0.5
	Plant-based Spoonable Yoghurts	2	2.5	2.5-2.5	0
	Plant-based drinks	29	4	1.5-5	0.5
	Rice/nut/grain & Seed Based Drinks	2	4	4-4	0
	Soy-based Drinks	2	4.75	4.5-5	0.5
	Butter	8	0.5	0.5-1	0
	Fresh Cheese & Cream Cheese	2	1	1-1	0
	Cream	9	1	0.5-2	1
	Margarine & Other Blends	3	3	3-3.5	0.5
	Curd & Quark	4	5	1.5-5	1.75
	Evaporated Milk	3	2	1.5-4.5	3
	Sweetened Condensed Milk	2	1	1-1	0
	Creamers	1	0.5	0.5-0.5	0
**Desserts & Ice Cream**		**159**	**2.5**	**0.5-5**	**1.5**

	Dairy Based Ice Cream & Frozen Yogurt	49	2	0.5-4.5	1
	Chilled Desserts	25	1.5	0.5-4	0.5
	Shelf-Stable Desserts	55	3.5	2-5	1
	Frozen Desserts	8	1.5	1-3	1
	Plant Based Ice Cream & Frozen Yogurt (Dairy Alternatives)	1	3	3-3	0
	Dessert Toppings	17	2	0.5-5	2
	Water Based Ice Lollies, Pops & Sorbets	4	3.25	3-4	0.75
**Fruit & Vegetables**		**217**	**4.5**	**3-5**	**1**
	Vegetables	159	5	3-5	0.5
	Fruit	58	4	3-5	1
**Hot Beverages**		**16**	**3.5**	**0.5-5**	**2.5**
	Tea	1	0.5	0.5-0.5	0
	Coffee	6	2.75	1.5-3.5	2
	Malt & Other Hot Beverages	9	4	1.5-5	2.5
**Juice Drinks**		**173**	**5**	**0.5-5**	**2.5**
	Juice	150	5	4-5	0
	Nectars	22	1.5	0.5-5	3.5
	Fruit/Flavoured Still Drinks	1	1.5	1.5-1.5	0
**Meals & Meal Centres**		**475**	**3.5**	**1.5-5**	**1**
	Prepared Meals	274	3.5	2-4.5	0.5
	Meal Kits	42	3.5	1.5-5	0.5
	Pastry Dishes	52	3	1.5-3.5	0.5
	Salads	38	4	2.5-4.5	0.5
	Pizzas	36	3	2-3.5	0.5
	Instant Noodles	17	3	2-3.5	0
	Sandwiches/Wraps	3	3.5	2-4	2
	Instant Rice	4	3.5	3-3.5	0
	Instant Pasta	9	3.5	3-3.5	0
**Other Beverages**		**40**	**4**	**1-5**	**2.5**
	Meal Replacements & Other Drinks	27	4.5	2-5	1.5
	Beverage Concentrates	7	1.5	1-3.5	1.5
	Beverage Mixes	6	4.5	1.5-4.5	2
**Processed Fish, Meat & Egg Products**		**516**	**3.5**	**0.5-5**	**1**
	Meat Products	199	3	0.5-4.5	2.5
	Poultry Products	116	3.5	2-4.5	0.5
	Fish Products	179	4	1.5-4.5	0.5

	Eggs & Egg Products	7	4	4-4	0
	Meat Substitutes	15	4	3-5	1
**RTDs**		**17**	**3**	**2-5**	**1.5**
	Iced Coffee	12	3.25	2.5-5	1
	Iced Tea	5	2.5	2-2.5	0.5
**Sauces & Seasonings**		**252**	**3**	**0.5-5**	**1.75**
	Seasonings	23	3.5	0.5-5	3.5
	Cooking Sauces	78	3	0.5-5	1.5
	Oils	30	3.75	0.5-4.5	1
	Table Sauces	17	1.5	0.5-4	1
	Dressings & Vinegar	12	1.5	1-2	0.75
	Pickled Condiments	16	3	1.5-5	0.75
	Pasta Sauces	40	4	1-4	1
	Stocks	26	3	2.5-3.5	0
	Mayonnaise	4	1.5	1.5-4	1.25
	Other Sauces & Seasonings	6	4.5	2-5	1
**Savoury Spreads**		**24**	**3.5**	**1-4.5**	**1.5**
	Dips	19	3.5	1-4.5	1.5
	Meat Pastes & Pates	5	2.5	2-3.5	1
	Savoury Vegetable Pastes/Spreads				
	Yeast Extracts				
	Sandwich Fillers/Spreads				
**Side Dishes**		**208**	**4**	**2-5**	**1**
	Pasta	64	4	2-5	0.75
	Rice	42	3.5	2.5-4.5	0.5
	Potato Products	63	4	3-4.5	0.5
	Stuffing, Polenta & Other Side Dishes	31	4	3-5	1
	Noodles	8	4	3.5-4	0.5
**Snacks**		**657**	**4**	**0.5-5**	**1.5**
	**Snack/Cereal/Energy Bars**	224	4	1-5	1
	**Hors d'oeuvres/Canapes**	89	2.5	0.5-4	1
	Nuts	105	4.5	1.5-5	1
	Fruit Snacks	60	4	1.5-5	1
	Potato Snacks	22	2.5	1-3.5	1
	Corn-based Snacks	22	3.5	0.5-4.5	2.5
	Rice Snacks	13	3	1-3.5	1.5
	Snack Mixes	41	4	1-5	1
	Vegetable Snacks	27	5	2.5-5	0
	Popcorn	13	4	1-5	2.5

	Wheat & Other Grain-based Snacks	32	4	3.5-5	1
	Bean-based Snacks	8	3.75	2.5-4	1
	Meat Snacks				
	Other Snacks				
	Cassava & Other Root-based snacks	1	3	3-3	0
**Soup**		**139**	**3.5**	**2-4**	**0.5**
	Wet Soup	101	3.5	2-4	0
	Dry Soup	38	3.25	3-3.5	0.5
**Sports & Energy Drinks**		**1**	**1.5**	**1.5-1.5**	**0**
	Sports Drinks				
	Energy Drinks	1	1.5	1.5-1.5	0
**Sugar & Gum Confectionary****		70	1.5	0.5-2.5	0.5
**Sweet Spreads**		**52**	**4**	**0.5-5**	**4**
	Honey	8	1	1-1	0
	Nut Spreads	29	5	3.5-5	0.5
	Chocolate Spreads	7	1	0.5-5	3.5
	Confiture & Fruit Spreads	4	1.75	1.5-2	0.5
	Syrups	2	1	1-1	0
	Caramel & Cream Spreads	2	0.75	0.5-1	0.5
**Sweeteners & Sugar**		**4**	**.5**	**0.5-2**	**0.75**
	Sucrose	3	0.5	0.5-0.5	0
	Other Natural Sweeteners	1	2	2-2	0
	Artificial Sweeteners				
**Water**		**22**	**2**	**2-5**	**3**
	Flavoured Water	13	2	2-2.5	0
	Water	9	5	5-5	0
**Total**		**4251**	**3.5**	**0.5-5**	**1.5**

* Mintel Sub-categories available but granular data not relevant for this category; Categories are defined by Mintel and relate to product similarities, and do not correspond with NOVA and Australian Dietary Guideline criteria.

Scheme S1. Australian Dietary Guidelines classification rules.

Coding categories:

1-grain foods;

2-fruit;

3-vegetables;

4-meat/eggs/tofu/nuts/seeds/legumes;

5-milk/yoghurt/cheese/alternatives;

6-mixed meals or food mixes consistent with ADGs FFGs;

7-discretionary foods;

8-culinary ingredients: oils, margarine, liquid stock, cocoa, baking powder etc.

9-formulated foods; and
10-water.

ADGs Classification Sequence:

- First determine if a food product can be classified as discretionary (1), culinary (2), formulated (3), or water (4). If not classified into one of these four categories, the food product is a five food group food (5), and rules outlined under bullet point five should be followed.

1. Discretionary Foods

A product is classified as discretionary if:

1.1 The product corresponds to a food or beverage flagged as discretionary in the ABS's *Discretionary Food List* at the unique code level (8-digit code). For unique or novel food products that do not appear in AUSNUT but are potentially discretionary, an item which closely resembles the novel product with regards to nutritional composition, ingredients, and purpose can be used as a proxy (with consensus amongst researchers).

1.2 The product is a breakfast cereal with sugar content over 30g per 100g, or over 35g per 100g if the breakfast cereal contained fruit. This is based on criteria outlined in the ABS's *Principals for Identifying Discretionary Foods*.

1.3 The product is a fruit, nut or seed-based snack bar or ball flagged as discretionary in the *Discretionary Food List* that does not meet criteria 5.5.

1.4 The product is a savoury cereal-based biscuit or snack that does not correspond to an item at the unique coded level in AUSNUT, and has an energy content over 1800kj per 100g. This is based on criteria for savoury biscuits at the 5-digit code level in the *ABS's Discretionary Food List*.

1.5 The product can be classified as a mixed food (consists of more than two food groups with no one food group at a proportion over 50%), and is not flagged on the *Discretionary Food List,* but has a saturated fat content over 5g per 100g. This is based on ABS's criteria for mixed dishes with cereal content in the ABS's *Principals for Identifying Discretionary Foods*, but the rule has been extended to include all meals due to the difficulty in classifying some products not present in AUSNUT. The criteria applies specifically to meals, not to food mixes consisting of nuts, seeds, and fruit.

1.6 The product is a mixed food (consisting of more than two food groups with no one food group at a proportion over 50%), and is not flagged on the *Discretionary Food List,* but over 50% of the product consists

of sauce. This is based on the *ABS's Discretionary Food List* classification – all commercial sauces, including pasta sauces, curry pastes and simmer sauces, are flagged as discretionary. The criteria does not apply to soups, as they largely consist of water.

1.7 The food product is a ready-to-heat pizza and either: the pizza base thickness cannot be determined to enable the identification of the correct corresponding item in AUSNUT (i.e. when the discretionary classification depends on base-thickness for the particular pizza topping type, base is assumed to be thin); or the product does not correspond to item in AUSNUT and saturated fat content is over 5g per 100g.

1.8 The product is a small portion of savoury food coated in a crumb or batter (e.g. chicken nugget, arancini, or cheese ball).

1.9 The food product contains alcohol as a flavouring but is not a cooked food.

1.10 If classification is not straightforward using criteria 1.1-1.9 a decision needs to be made by discussion and consensus among all researchers. These decisions should be documented.

2. Culinary Ingredients

The food product is a single ingredient item used in cooking that cannot be classified as FFG or discretionary (i.e. it cannot be classified into one the five groups as visually represented in the *Australian Guide to Healthy Eating*, and is not flagged as discretionary in the ABS's *Discretionary Food List*). Food products that should be classified as culinary include: cooking oils, margarine, vinegar, baking powder, ground spices, cocoas, and liquid stock.

3. Formulated Foods

The food product has the words FORMULATED SUPPLEMENTARY FOOD displayed on the label (as defined in Food standard 2.9.3). These products are not intended to display an HSR and include formulated supplementary foods for young children, formulated supplementary sports foods, and foods for special medical purposes. *(Infant formulas, infant foods, and alcohol are also not intended to display an HSR, but these products have been excluded in the data extraction step)*

4. Water

Only unflavoured bottled water and sparkling mineral water with no energy content, not flagged as discretionary in the ABS's *Discretionary Food List*.

5. Five Food Group foods

5.1 Products are classified into one of the five food groups based on the way foods have been grouped in the *Australian Guide to Healthy Eating*.

5.2 A five food group food must contain over 50% of a food belonging to that group. This was on the basis that the product would provide a substantial proportion of the intake for that food group.

5.3 Mixed foods were defined as products that contained ingredients from two or more food groups, with no one food group making up over 50% of the composition. These are products consistent with ADGs but cannot be categorised into one of the five groups due to mixed content.

5.3.1 Where percentages for ingredients cannot be determined, but there are clearly two or more food groups, the food product is a mixed food.

5.3.2 Soup products containing more than two food groups are always classified as mixed foods due the difficulty in determining the proportion of each food group.

5.4 Legumes are classified as both meat and vegetables in the *Australian Guide to Healthy Eating*. For consistency in classification, legumes products that are likely to be used in meals as meat alternatives should be classified as meat, and all other legumes products should be classified as vegetables.

5.5 For mixed foods that contain legumes and meat, the ingredients can be combined to meet the 50% proportion required to be classified as meat. For mixed foods that contain legumes and vegetables, the ingredients can be combined to meet the 50% proportion required to be classified as vegetables.

5.6 Bars or balls consisting predominantly of dried fruit, nuts or seeds with no added sugars are classified as five food group foods. This criterion was created to classify foods that were essentially dried fruit or nuts, given the large number of these types of products displaying HSRs.

- If the food cannot be classified into categories 7-10 using the outlined methods, it is considered a five food group food, and should be classified after discussion and consensus among the three researchers.
